# Scoring disease in an animal model of multiple sclerosis using a novel infrared-based automated activity-monitoring system

**DOI:** 10.1038/s41598-019-55713-7

**Published:** 2019-12-16

**Authors:** Shailesh K. Shahi, Samantha N. Freedman, Rachel A. Dahl, Nitin J. Karandikar, Ashutosh K. Mangalam

**Affiliations:** 1 Department of Pathology, University of Iowa, Iowa City, IA 52242 USA; 2 Interdisciplinary Graduate Program in Immunology, University of Iowa, Iowa City, IA 52242 USA; 3 Graduate Program in Molecular Medicine, University of Iowa, Iowa City, IA 52242 USA; 4 Carver College of Medicine, University of Iowa, Iowa City, IA 52242 USA

**Keywords:** Autoimmunity, Neuroimmunology

## Abstract

Multiple sclerosis (MS) is a chronic demyelinating disorder of the central nervous system (CNS). Its corresponding animal model, experimental autoimmune encephalomyelitis (EAE), is widely used to understand disease pathogenesis and test novel therapeutic agents. However, existing methods to score EAE disease severity are subjective and often vary between individual researchers, making it difficult to translate findings across different studies. An enhanced automated method of disease scoring would eliminate subjectivity and reduce operator-dependent errors. Here, we used an **I**nfra-**R**ed **A**ctivity **M**onitoring **S**ystem (IRAMS) to measure murine locomotor activity as a surrogate measure of disease severity and compared it to standard EAE scoring methods. In mice immunized with CNS-specific myelin antigens, we observed an inverse correlation between disease severity and mouse activity, with the IRAMS showing enhanced disease scoring compared to standard EAE scoring methods. Relative to standard EAE scoring methods, IRAMS showed comparable measurement of disease relapses and remissions in the SJL/J-relapsing-remitting model of EAE, and could comparably assess the therapeutic efficiency of the MS drug, Copaxone (Glatiramer acetate-GA). Thus, the IRAMS is a method to measure disease severity in EAE without subjective bias and is a tool to consistently assess the efficacy of novel therapeutic agents for MS.

## Introduction

Multiple sclerosis (MS) is a chronic inflammatory and demyelinating disorder of the central nervous system (CNS), which results in significant morbidity and can lead to mortality. Collective evidence suggests that an important hallmark of MS is the onset of disease due to a T-cell-mediated aberrant immune response directed against a number of CNS-specific myelin antigens. Due to variable efficacy among current disease-modifying therapies, there is need for the development of better treatment options.

Experimental autoimmune encephalomyelitis (EAE) is the most frequently used animal model to study the immunopathogenesis of MS^1–4^, and to test the therapeutic efficacy of novel agents. Numerous therapeutic strategies have utilized the EAE model to assess the efficacy and toxicity of novel drugs. As such, this model has attained a pivotal role in understanding the pathogenesis of disease as well as developing novel therapeutics for MS^1–4^, as illustrated by the number of approved therapies for MS, including: Glatiramer acetate, Mitoxantrone, Natalizumab, and Ocrelizumab^5–7^.

EAE can be induced in various inbred animal strains by inoculating the animal with whole myelin or defined myelin protein with adjuvants^8,9^, which leads to activation of autoreactive peripheral CD4 T-cells and their subsequent trafficking to the CNS by transgressing the blood brain barrier. Typically, between 7–12 days after immunization, infiltrating inflammatory cells attack the myelin sheath, resulting in motor deficits and ascending paralytic disease. These defects are quantified using a standard EAE scoring system on a 0–5 disease severity scale: 0, no disease; 1, loss of tail tone; 2, hind limb weakness; 3, hind limb paralysis; 4, hind limb paralysis and forelimb paralysis or weakness; and 5, moribund/death^10^. While standard scoring of EAE disease severity on a 0–5 numerical scale is commonly used by scientists all over the world, there is a likelihood of operator/assessor-dependent variability as the clinical scoring between different researchers is highly subjective. In addition, this standard scoring system requires the daily handling of animals, the stress of which alters corticosterone levels and confounds EAE disease progression^11^. Furthermore, animals with similar scores might have nuanced motor deficits which may not be appreciated by daily observation.

To overcome the limitations of the current scoring system, including operator independent/dependent challenges, we utilized **Opto-M4 I**nfra-**R**ed **A**ctivity **M**onitoring System (IRAMS) (Columbus Instruments International, Columbus, OH) as an alternative method to score disease severity in EAE. Rearing activity, i.e. standing on their hind limbs to reach higher elevation, is a primordial animal behavior in which healthy mice aim to reach higher elevation to explore and draw environmental cues. We utilized IRAMS to assess mouse rearing activity as a surrogate marker for EAE disease progression and severity, which detected comparable disease activity scores as those obtained using standard EAE scoring methods. Additionally, IRAMS enabled us to record disease relapses and remissions in a model of relapsing-remitting EAE (RR-EAE). We also used IRAMS to evaluate the effect of Copaxone, a well-established MS drug, in suppressing disease in a preclinical model of MS^12,13^. Our results suggest that infrared-based monitoring of nocturnal activity provides an improved assessment of quantitative motor deficits and ambulatory function and can be used as a surrogate to current scoring systems to detect disease severity in murine models of MS. This advancement in detection systems enables the field to reduce operator-dependent subjective errors and minimizes the impact of daily human handling of mice, thus nullifying potential bias and human-induced stress that can affect disease severity.

## Results

### Establishing the optimal time interval and day *vs* night time for measuring mouse activity using the IRAMS

**Figure 1:**
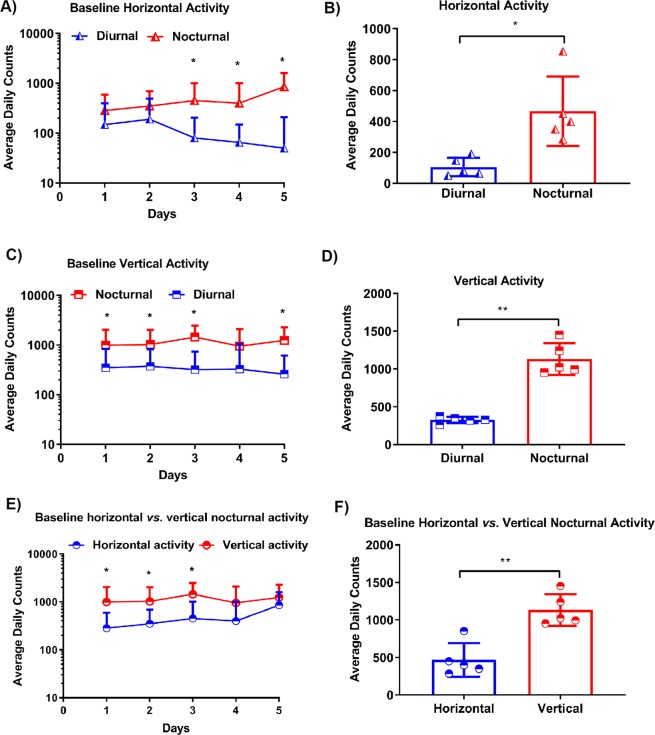
Baseline average spontaneous activity (horizontal and vertical) of HLA-DR3.DQ8 Tg mice during the day (diurnal) and night (nocturnal). (**A**) Average daily counts of spontaneous diurnal and nocturnal horizontal activity of healthy HLA-DR3.DQ8 Tg mice. Measurements were obtained for 24 hours up to day 5. (**B)** Average daily counts of 5 days spontaneous diurnal and nocturnal horizontal activity of healthy HLA-DR3.DQ8 Tg mice. (**C)** Average daily counts of spontaneous diurnal and nocturnal activity of healthy HLA-DR3.DQ8 Tg mice. Measurements were obtained for 24 hours up to day 5. (**D)** Average of 5 day counts of spontaneous diurnal and nocturnal activity of healthy HLA-DR3.DQ8 Tg mice. (**E)** Comparison of the average daily counts of spontaneous horizontal and vertical nocturnal activity of healthy HLA-DR3.DQ8 Tg mice for 24 hours up to day 5. (**F)** Comparison of the average of 5 day counts of spontaneous horizontal and vertical nocturnal activity of healthy HLA-DR3.DQ8 Tg mice. For all panels, the data represent one experiment out of three performed. (n ≥ 7 mice per group). The *p* values in A, C, E and F were calculated using an unpaired t test with two step-up methods of Benjamini, Krieger and Yekutieli. The *p*-value in B was calculated using an unpaired t test with Welch’s correction, and that in D was calculated using an unpaired t test using the Kolmogorov-Smirnov test. *indicates *p* ≤ 0.05, ** indicates *p* < 0.005 when comparing diurnal *vs*. nocturnal baseline activity.

To determine the time interval needed to capture optimal activity measurements using IRAMS, mouse activity within a cage was recorded for 24 h ×5 day [during every day (diurnal) and night (nocturnal) at 10, 20, 30, and 60 minutes]. We placed Human Leukocyte Antigen (HLA)-DR3.DQ8 transgenic (Tg) mice (8–12 weeks old) in their normal cages, and placed these within IRAMS brackets equipped with two sensors (Columbus Instruments, Columbus, OH) (Supplemental Fig. S1) to measure both horizontal (X-axis) and vertical (Z-axis) activity during day (diurnal − 6.01 AM to 6.00 PM) or night (nocturnal − 6.01 PM to 6.00 AM). Activity measurements at 30 minutes showed the maximum activity for both horizontal (Fig. 1A,B) and vertical movement (Fig. 1C,D), and was similar to activity measurements at 10 or 20 minutes. In contrast, measurements at 60 minutes reported less activity compared to measurements at any other time point. As expected, we observed that mice displayed a greater amount of horizontal and vertical nocturnal activity relative to diurnal activity. Horizontal counts were higher at night (range 285–854) compared to day time counts (range 50–190) **(**Fig. 1A,B and Table 1**)**. Vertical diurnal counts ranged from 260–375 in contrast to nocturnal counts of 954–1452 **(**Fig. 1C,D and Table 1**)**. These data indicate that nocturnal assessments record optimal mouse activity. Thus, we chose nocturnal 30 minutes as the optimal time interval to assess activity measurements using IRAMS.

**Table 1:** Average spontaneous activity (horizontal *vs*. vertical) of healthy mice during the day (diurnal) and night (nocturnal).

	Minimum Values (count)	Maximum Values (count)	Mean ± SD
**Horizontal Activity**			
Diurnal	50	190	106.6 ± 59.81
Nocturnal	285	854	467.6 ± 224.4
**Vertical Activity**			
Diurnal	260	375	326.7 ± 42.9
Nocturnal	954	1452	1133 ± 209.9
**Nocturnal Activity**			
Horizontal	285	954	467.6 ± 224.4
Vertical	854	1452	1133 ± 209.9

Statistical analyses were done with GraphPad Prism 7.

Next we measured both horizontal and vertical activity for 30 minute intervals within a 24 hour cycle to determine whether diurnal (6.01 AM to 6.00 PM) or nocturnal (6.01 PM to 6.00 AM) measurements similarly reflect animal activity **(**Fig. 1E,F**)**.

Measurements of either nocturnal horizontal or vertical activity revealed that horizontal measurements gave an average of 467.6 ± 224.4 counts, whereas vertical measurements gave a count of 1133 ± 209.9 **(**Fig. 1E,F and Table 1**)**. Thus, our data indicated that a nocturnal measurement of vertical activity at 30-minute intervals was optimal for monitoring mouse activity using IRAMS. Hence, we used these parameters to measure disease activity in our EAE animal model.

### Monitoring disease activity using the IRAMS

**Figure 2:**
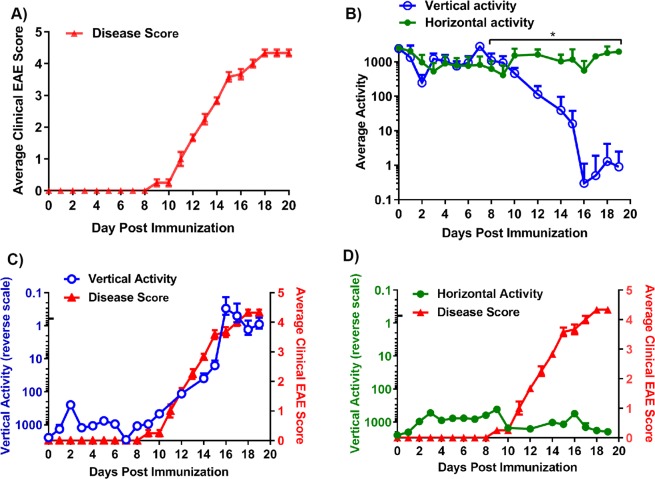
Clinical EAE scores and spontaneous vertical activity show a similar pattern of disease severity in HLA-DR3.DQ8 Tg mice induced with EAE. (**A)** Average clinical EAE score in HLA-DR3.DQ8 Tg mice immunized with PLP_91–110_ on day 0. Mice were assessed daily using standard EAE scoring for 20 days post-immunization. (**B)** Average daily counts of spontaneous horizontal and vertical activity of mice in A. Measurements of daily average nocturnal activity (6:01 PM to 6:00 AM) were obtained up to day 19. *P* value determined by Mann-Whitney unpaired t test. (**C)** Plots of the average clinical EAE score (right Y-axis) and spontaneous vertical activity (inverted log10 on left Y-axis) of mice in (**A**). (**D)** Average clinical EAE score (right Y-axis) and spontaneous horizontal activity (inverted log10 in left Y-axis) of mice in (**A**). For all plots, the data presented represent one of three experiments performed (n ≥ 7 mice per group).

We have previously utilized humanized HLA class II Tg mice as an animal model of human MS^13,14^. To obtain baseline counts prior to disease induction, nocturnal vertical (Z-axis) and horizontal activity (X-axis) at 30-minute intervals was measured every day up to day 5 **(**Fig. 1**)**. To induce disease, HLA-DR3.DQ8 Tg mice were immunized with PLP_91–110_ peptide emulsified in complete Freund′s adjuvant (CFA) containing 400 µg of *Mycobacterium tuberculosis* H37Ra (MTb) (Becton, Dickinson and Company, Sparks, MD, USA) and 80 ng Pertussis toxin (PTX) (Sigma Chemicals, St. Louis, MO, USA), was given i.p. on day 0 and day 2 post-immunization. We compared disease severity as measured using the standard EAE scoring system (0–5 scale) and the IRAMS. Mice that were immunized with the PLP_91–110_ peptide had increased clinical EAE scores beginning on day 9 post-immunization, and ascending paralytic disease starting with tail atony began on days 9–10, followed by hind limb weakness (days 11–14), and full blown paralytic disease by day 11, with moribund animals sacrificed on day 20 post-immunization (Fig. 2A). Measurements obtained using the Opto-M4 IRAMS showed a decrease in vertical activity counts on day 1 (1310 ± 446 vs 954 ± 540) and 3 (1355 ± 1132 vs 254 ± 292) post-immunization compared to baseline activity counts (Fig. 2B), with the decline in activity being greater on day 3 compared to day 1 post-immunization. All animals recovered from this lapse in activity and gained some vertical activity over the next 4 days. However, on day 8 post-immunization, mice began to show a decline in vertical activity and a maximum drop in vertical activity was observed by day 16, with the majority of animals showing minimal vertical activity from days 10 to 16 (Fig. 2B). Notably, there was a slight gain in vertical activity from days 16 to 18, which was followed by a slight loss of vertical activity for the following two days (Fig. 2B). By day 20 post-immunization, animals had not recovered from the disease and had to be sacrificed due to their moribund state. Thus, detection of vertical activity using the IRAMS indicated early signs of disease (i.e., reduced vertical activity) at day 8 post-immunization, whereas standard EAE scoring methods only detected early disease starting at day 9/10. In addition, vertical activity monitoring showed at least one relapsing-remitting disease episode (remitting at 16 and relapsing at day 19). Notably, a similar pattern was observed when we plotted the spontaneous vertical activity of PLP_91–110_-immunized mice on a reverse scale along with the average clinical EAE score (Fig. 2C). In contrast, there was no similarity between the spontaneous horizontal activity plotted on a reverse scale compared to the average clinical EAE score (Fig. 2D). Thus, our data indicate that vertical activity measurements are capable of identifying subtle changes in motor dysfunction that are indicative of disease.

**Figure 3:**
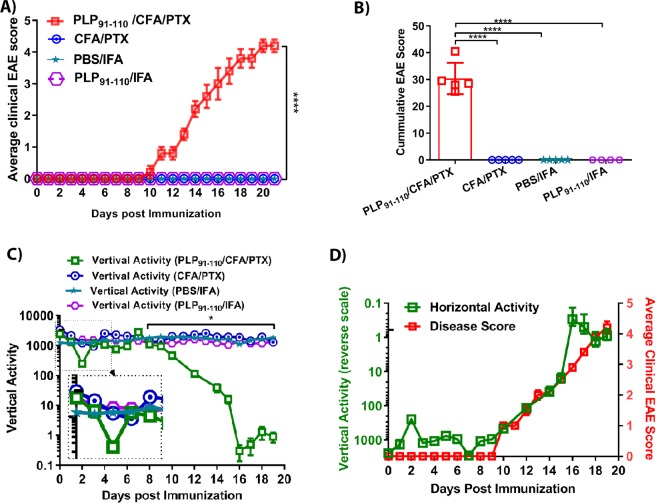
Changes in mouse activity are specific for induction of EAE disease. (**A)** Average clinical EAE score of HLA-DR3.DQ8 Tg mice immunized with PBS/IFA alone, CFA/PTX alone, PLP_91–110_ with CFA/PTX, or PLP_91–110_ with IFA. Mice were immunized on day 0 and assessed daily by standard disease scoring for 20 days post-immunization. (**B)** Cumulative EAE score of HLA-DR3.DQ8 Tg mice immunized as in (**A**). (**C)** Average spontaneous vertical activity of HLA-DR3.DQ8 Tg mice immunized as in A. Measurements of daily average nocturnal activity (6:01 PM to 6:00 AM) were obtained up to day 19. (**D)** Average clinical EAE score (right Y-axis) and spontaneous vertical activity (inverted left Y-Axis) of HLA-DR3.DQ8 Tg mice immunized as in A. The data presented represent one of three experiments performed (n ≥ 5 mice per group). *p*-values for average clinical EAE score (**A**) and vertical activity analysis (**C**) determined by one-way ANOVA with Dunnett’s multiple comparisons test; and for cumulative EAE score (**B**) by Mann-Whitney unpaired t test. * indicates *p* ≤ 0.05; *** indicates *p* ≤ 0.0002; **** indicates *p* < 0.0001.

In our model of EAE, disease is induced by injecting mice with an emulsion that includes CFA and PTX as adjuvants that are mixed with myelin PLP antigens. Therefore, we determined if these adjuvants had any effect on vertical mouse activity and thus contributed to the changes that were observed. Mice were divided into four groups: 1) mice that received CFA + PTX; 2) mice that received PLP_91–110_ + CFA + PTX; 3) mice that received PBS + IFA, and PLP_91–110_ + IFA. Disease severity was monitored using both standard EAE scoring method and the IRAMS. Only mice that were immunized with PLP_91–110_ + CFA + PTX developed clinical EAE disease, whereas no clinical disease was observed in other groups that received CFA + PTX. PBS + IFA (phosphate buffer saline-PBS in Incomplete Freund′s adjuvant -IFA), or PLP_91–110_ + IFA (Fig. 3A,B). Monitoring vertical activity revealed that mice that received either PLP_91–110_ + CFA + PTX or CFA + PTX had decreased activity on the days immediately following immunization (i.e., days 1 and 3 post-immunization) and began to recover starting on days 3–4, whereas only mice that received PLP_91–110_ + CFA + PTX began to show a decrease in vertical activity as well as ascending paralysis starting on day 8 post-immunization (Fig. 3C). The mice that received PBS + IFA or PLP_91–110_ + IFA maintained a similar level of vertical activity throughout the duration of the experiment and mice that received CFA + PTX showed no decline in vertical activity following the initial dip at days 1 and 3 (Fig. 3C). As observed previously, plotting the spontaneous vertical activity of mice with PLP_91–110_-induced EAE on a reverse scale showed a similar pattern as the graph of the average clinical EAE score (Fig. 3D). Thus, measuring vertical activity using the IRAMS detected minimal and subtle changes in disease with reduced variations due to automated scoring. The lack of significant changes in activity of mice in the control groups showed that changes in vertical activity are due to EAE disease induced by the PLP-antigen and are not due to the effects of the adjuvant.

### The IRAMS detects disease activity in a relapsing-remitting (RR)-EAE disease model

**Figure 4:**
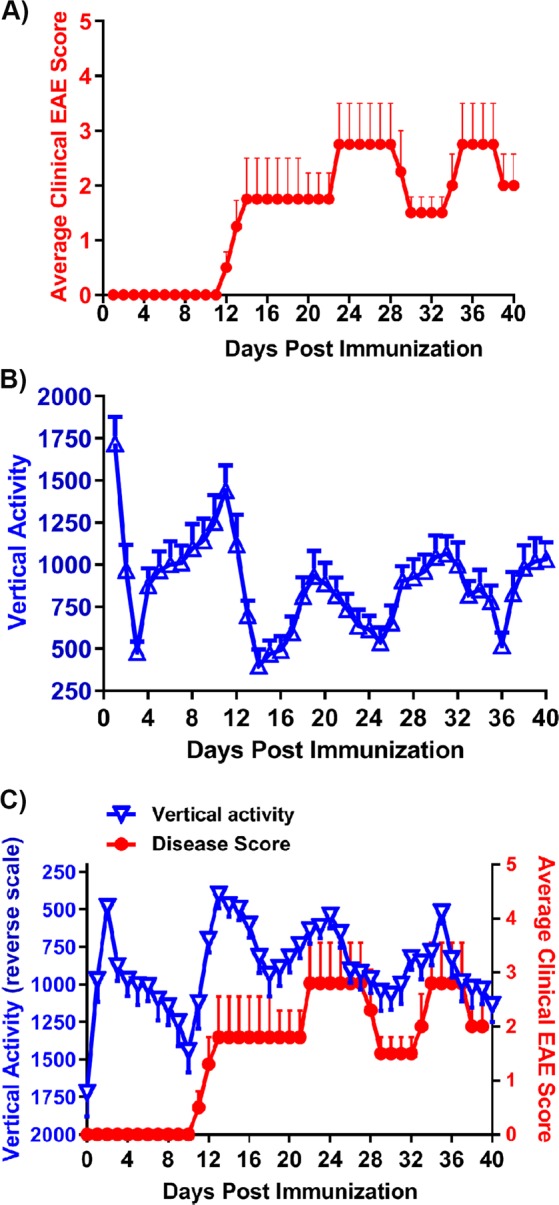
Disease severity as detected by changes in mouse activity is similar to standard scoring in RR-EAE. (**A)** Average clinical EAE score of SJL/J mice immunized with PLP_139–151_. Mice were immunized on day 0 and were assessed daily using standard disease scoring for 40 days post-immunization. (**B)** Average spontaneous vertical activity of SJL/J mice immunized as in A. Measurements of daily average nocturnal (6:01 PM to 6:00 AM) activity were obtained up to day 19. (**C)** Average clinical EAE score (right Y-axis) and spontaneous vertical activity (inverted left Y-axis) of SJL/J mice immunized as in A. For all plots, data represent one experiment out of three performed (n ≥ 5 mice per group).

As the disease in HLA-DR3.DQ8 Tg mice is typically observed to be monophasic and chronic, we also tested the ability of the IRAMS to measure RR-EAE. SJL/J mice immunized with PLP_139–151_ typically exhibit relapsing-remitting disease, which is characterized by multiple episodes of recovery from disease (remission) followed by disease relapse^15^. After the initial peak of disease, relapses are separated by states of remission, characterized by small changes in disease score for a short duration (few days). Using standard EAE scoring methods, disease scores can fluctuate between 1.5–4.0; thus, we reasoned that the IRAMS would be able to identify subtle changes in motor function that would otherwise not be reflected in the disease score. Baseline nocturnal vertical activity was measured in female SJL/J mice prior to immunization with PLP_139–151_ antigen emulsified in CFA. Following immunization, disease severity was assessed using both the standard EAE scoring method and the IRAMS. Using the standard EAE scoring system, we observed the onset of disease on day 11, with the peak of disease observed at day 22 post-immunization (Fig. 4A). We observed the first disease remission at day 29 and the first disease relapse at day 33, followed by another, milder remission around day 38 (Fig. 4A). In contrast using the IRAMS, we first observed a decline in vertical activity at days 10–11 post-immunization with the peak of disease observed at day 14 (Fig. 4B). Mice induced with EAE started to show signs of recovery as the vertical activity began to increase starting on day 15, and continued increasing until day 20 post-immunization (Fig. 4B). Starting on day 21 post-immunization, these mice showed a gradual loss of activity indicative of a state of relapse, which lasted for 4 days and was followed by another recovery phase characterized by a continuous gain in vertical activity. This recovery phase lasted for 5 days, after which animals again experienced a relapsing episode characterized by a loss of vertical activity, with mice showing only minimal activity at day 37. This disease relapse was followed by another recovery phase and no further disease relapse was observed for the duration of the experiment (Fig. 4B). With the use of the IRAMS, we detected three states of remission and two periods of relapse, whereas only two states of remission and one period of relapse were observed using standard EAE scoring (Fig. 4C). Thus, our data suggest that IRAMS is more sensitive in detecting episodes of disease remission and relapse compared to standard EAE scoring methods.

### The IRAMS can detect the therapeutic efficacy of Copaxone on EAE disease

**Figure 5:**
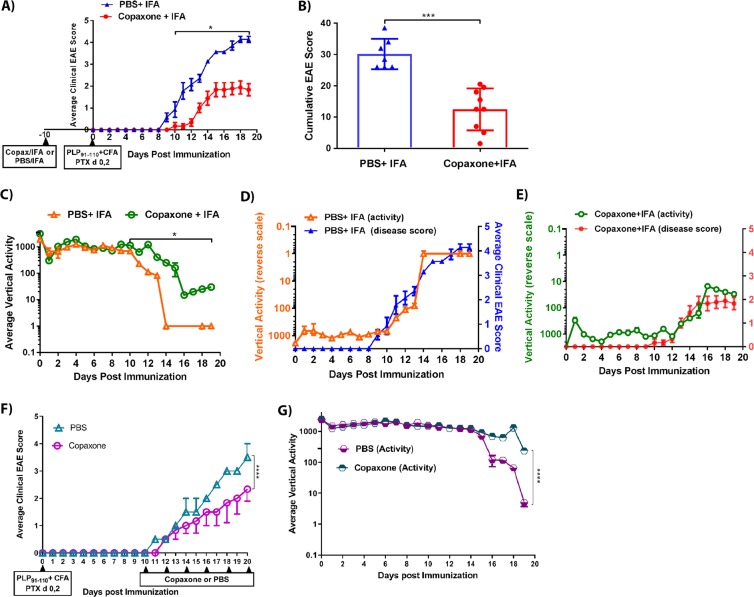
Changes in mouse activity can accurately detect the effects of prophylactic and therapeutic treatment of Copaxone in mice induced with EAE. (**A)** Average clinical EAE score of HLA-DR3.DQ8 Tg mice immunized on day 0 with PLP_91–110_ with CFA/PTX and treated with Copaxone or PBS on day −10. Mice were assessed daily using standard disease scoring for 20 days post-immunization. (**B)** Cumulative EAE score of HLA-DR3.DQ8 Tg mice in A. (**C)** Average spontaneous vertical activity of HLA-DR3.DQ8 Tg EAE mice in A. Measurements of daily average nocturnal activity were obtained up to day 19. (**D)** Average clinical EAE score (right Y-axis) and spontaneous vertical activity (inverted left Y-axis) of mice in A that were only treated with PBS. (**E)** Average clinical EAE score (right Y-Axis) and spontaneous vertical activity (inverted left Y-axis) of mice in A that were only treated with Copaxone. (**F)** Average clinical EAE score of HLA-DR3.DQ8 Tg mice immunized on day 0 with PLP_91–110_ with CFA/PTX and treated with Copaxone or PBS on day 10. Mice were assessed daily using standard EAE disease scoring for 20 days post-immunization. (**G)** Average spontaneous vertical activity of HLA-DR3.DQ8 Tg EAE mice treated with Copaxone or PBS as in F. Measurements of daily average nocturnal activity were obtained up to day 19. For plots (**A–E**), the data presented represent one of three experiments performed (n ≥ 7 mice per group). *p*-values determined by multiple t tests using the Holm-Sidak method (**A**) and the Mann- Whitney unpaired t test (**C**). * indicates p ≤ 0.05, when compared to the PBS-challenged group. For plots (**F,G**), *p*-values for average clinical EAE score (**F**) and vertical activity analysis (**G**) determined by one-way ANOVA with Dunnett’s multiple comparisons test. **** indicates *p* < 0.0001, when compared to the PBS-challenged group.

Next, we evaluated whether the IRAMS could provide an alternative and sensitive method to evaluate the prophylactic efficacy of potential disease-modifying MS drugs. We tested the ability of IRAMS to detect the effects of Copaxone (a first-line MS drug) in HLA-DR3.DQ8 Tg model of MS. Tg mice were first treated with Copaxone (2 mg in IFA/mouse) subcutaneously^12,13^. After 10 days of Copaxone treatment, EAE was induced as mentioned previously^12,13^. Using a standard EAE scoring method, mice that received Copaxone showed milder EAE disease compared to those treated with PBS + IFA (p < 0.005) **(**Fig. 5A,B and Table 2**)**. The IRAMS showed a small dip in activity in both groups of mice after immunization, followed by a gain in activity **(**Fig. 5C**)**. However, on days 8–9, control mice treated with PBS + IFA started showing a gradual loss of vertical activity, with all animals showing minimal activity at day 14 (Fig. 5C). In contrast, mice that received Copaxone treatment first started to show a decrease in activity on days 10–11, with all mice showing minimal activity on day 16 (Fig. 5C). Furthermore, mice that were treated with Copaxone showed a small recovery in activity, whereas mice in the control group were relatively immobile, showing little activity (Fig. 5C). Plotting the spontaneous vertical activity on a reverse scale along with the average clinical EAE score of both Copaxone and PBS-treated mice demonstrated that these methods detected a similar trend in disease severity **(**Fig. 5D,E).

**Table 2:** Effect of Copaxone on disease severity in HLA-DR3.DQ8 Tg mice immunized with PLP_91–110_.

Treatment	Disease Incidence (%)	Mean onset of disease ± SD	Mean EAE Score ± SD	Number of mice with maximum severity score					
				0	1	2	3	4	5
PBS-IFA	7/7 (100%)	9.8 ± 0.98	30.14 ± 4.87	—	—	—	—	6	1
Copaxone	9/8 (89%)	12.37 ± 1.59	10.29 ± 5.95	1	2	4	2	—	—

Statistical analyses were done with GraphPad Prism 7.

Next, we evaluated whether the IRAMS could provide an alternative and sensitive method to evaluate the therapeutic effect of Copaxone. As with prophylactic treatment, therapeutic treatment of mice (1^st^ dose of Copaxone was administered 10 days post immunization) with Copaxone resulted in milder EAE disease compared to the group treated with PBS + IFA (Fig. 5F). The IRAMS showed that control mice treated with PBS + IFA have lower vertical activity compared with Copaxone treated mice (Fig. 5G).

Collectively, our data indicate that the IRAMS was as efficient at detecting EAE disease severity as standard EAE scoring methods (both on 0–5 scale as well as 0–10 scale). However, IRAMS had the advantage of being able to predict disease changes at an earlier time in prophylactic setting and require reduced handling of the mouse.

## Discussion

For any novel drug to reach clinical trial status, it needs to first be tested for safety and efficacy in an animal model of disease. EAE is a widely used pre-clinical animal model of MS^1–4^, and its significance is highlighted by the fact that the majority of drugs that are currently utilized to treat this disease have been developed with this model^16^. Classical EAE disease in mice is an ascending paralytic disease, with early visible symptoms being tail atony, which progresses to weakness/paralysis of hind limbs and forelimbs^17^. The disease is scored by manual assessment of mouse symptoms, which are ranked on a scale from 0–5^10^; however, this method of scoring is subjective to individual variability due to operator errors. The majority of therapeutic agents reduce disease severity by 0.5 to 1.5 points, but this is often accompanied by variation due to the subjective nature of the standard EAE scoring method, making it difficult to identify differences in disease treatments among individual research groups.

To overcome these challenges, we have developed a quantitative scoring system of EAE disease by taking advantage of the **Opto-M4 I**nfra-**R**ed **A**ctivity **M**onitoring **S**ystem (IRAMS), which is based on the inherent ability of mice to perform rearing activity. The advantages of IRAMS include the abilities to: (1) take multiple measurements in a single day without disruption to the natural environment; (2) measure nocturnal motor function, which is when mice are most active; (3) minimize the handling of animals, thus reducing stress and anxiety; (4) obtain quantitative measurements spread over a larger range (0–1000), which is in contrast to the narrow range of the standard scoring system (0–5) and provides a more accurate assessment of disease severity; (5) reduce experimental bias by obtaining multiple measurements spread over a 12 h period; and (6) measure the activity of multiple mice simultaneously. In addition, this system monitors disease activity in a normal mouse cage, thus reducing cost and eliminating the social isolation that is required for the majority of quantitative disease monitor systems. Thus, the IRAMS is a unique method to objectively quantitate EAE disease severity and has several advantages over standard EAE scoring methods.

In addition to the advantages listed above, the IRAMS utilizes infra-red sensing to detect locomotor changes in mice, monitoring both horizontal and vertical activity. Rearing activity, i.e. standing on their hind limbs to reach higher elevation, is a primordial animal behavior which allow mice to explore and draw environmental cues. Using mouse rearing as a surrogate marker for disease severity provides a more complete assessment of the state of health, as a normal mouse with full strength and normal motor function will perform multiple rearing activities at night. Detecting only horizontal activity would fail to capture changes in mouse rearing, and thus may give a false impression of the state of the severity of disease. Additionally, our data suggesting optimum activity measurements can be obtained at night, which is not surprising as mice are nocturnal animals. Thus, the IRAMS may provide a more accurate assessment of disease severity as most standard EAE scoring methods occur during the day.

To measure the utility of the IRAMS, we first used a chronic model of EAE in HLA class II Tg mice, which develop a chronic form of the disease similar to that observed in the C57BL/6-EAE mouse model^13^. The main objective of this study was to determine whether the IRAMS was as good at detecting EAE disease severity as a standard EAE scoring method. The rationale was that if a novel system can improve the quality and authenticity of result similar to or better than the existing 0–5 scoring system, it has the added advantages of providing unbiased quantitative multiple readings per day and reduced handling of animals. While the trends in disease were similarly detected with each method, both disease onset and the peak of disease could be detected slightly earlier with the IRAMS compared to standard EAE scoring methods. Interestingly, we noted a mild recovery in rearing activity using IRAMS, whereas the standard scoring system suggested disease was progressive with no recovery period. These data suggest that IRAMS can not only detect disease phenotypes earlier than standard scoring systems but can also identify periods of subtle recovery that are often missed by standard EAE scoring methods. In addition, we used adjuvant controls to rule out any confounding effects of the CFA and/or PTX on disease monitoring with the IRAMS. For the first two or three days post-immunization, only mice that received CFA + PTX showed effects on mouse activity that were similar to those observed in mice that received antigen plus CFA + PTX. However, after an initial dip in vertical activity, all animals that received CFA + PTX recovered completely and showed stable vertical activity for the duration of the experiment. It is well established that the combination of CFA and PTX leads to an initial cytokine storm, which could explain the initial reduction in vertical activity that we observed^2^. As some research labs use 10-point EAE scoring scale for measuring EAE^18^, we also compared IRMAS activity measurement with 10-point EAE score system. The correlation between disease activity and EAE disease on 10 point EAE score was similar to the observation made using 0–5 EAE scoring system (Supplementary Fig. S2). Overall our data validates that motor function/rearing activity measured by IRAMS can be used as a surrogate marker to monitor disease activity in an EAE model.

IRAMS was also able to detect disease relapses and remissions in a relapsing-remitting-EAE model in SJL/J mice, which mimics the relapsing-remitting clinical features of MS patients. IRAMS was able to detect three disease peaks, three disease remission episodes and two relapses. In contrast, standard EAE scoring methods only detected two disease peaks, two remissions, and one relapse. Thus standard scoring missed the initial period of remission in these mice. The first maximum decrease in activity lasted only for a day and mice recovered expeditiously from the loss of motor function; therefore it is likely that standard scoring failed to detect the first paralytic episode due to its short duration. However, IRAMS was able to detect a loss in activity due to the fact that a greater number of measurements were obtained (i.e., 13 measurements over 12 hr period with the IRAMS vs 1–2 measurements over a 24 hr period using standard EAE scoring methods). In SJL /J mice EAE score fluctuated between 1.5 to 3.5 and IRAMS was able to detect those small differences in disease score. This highlight the ability of IRAMS to detect subtle difference in disease score and suggests the usefulness of the system in evaluating therapeutic effects of novel compound even when difference between groups are moderate. These data reinforce the fact that the IRAMS is more sensitive in detecting disease severity compared to standard EAE scoring in a relapsing-remitting model of EAE.

To further validate the sensitivity of detecting disease severity using IRAMS, we tested its ability to assess the therapeutic efficacy of the MS drug Copaxone. We observed that IRAMS was able to detect a gain in motor function starting on day 17. In contrast we did not observe this gain in motor function using standard EAE scoring methods. This suggests that the higher sensitivity displayed by IRAMS enables us to detect even subtle differences in motor function.

Previously another study assessed locomotor activity combined with ultrasonic vocalizations, which suggested a protective effect of Fingolimod (FTY720) on EAE disease^19^. This required that a single mouse be placed in a special home-cage to enable automated measurements of movement and ultrasonic vocalizations. In contrast to this system, the IRAMS can measure mouse activity when mice are in their normal cage, and can measure the activity of 4–5 mice in each cage. Another study used a spontaneous activity monitoring system to show the therapeutic efficacy of a natural monoclonal human IgM antibody to preserve functional motor activity in a demyelinating disease model induced by Theiler’s murine encephalomyelitis virus^20^. This study used a special open field–rat configuration, which requires special housing and large spaces due to its big size. This differs from the IRAMS as this system requires less space and can measure both horizontal and vertical activity, as opposed to only vertical activity. Thus, we have refined the use of an activity monitoring system in order to obtain better measurements of disease activity while mice are in their natural environment, removing the need for large space or single-mouse housing.

Currently for those conducting biomedical research, there is an increased emphasis on the three Rs: replacement, reduction, and refinement. The IRAMS will help address two of these concepts as its use can reduce human handling of animals and also refine an existing standard procedure to score disease. In addition, monitoring activity using the IRAMS as a surrogate for disease severity will also minimize operator-based variability in EAE disease scoring. The use of an IRAMS requires up-front costs that are associated with the purchase of the system, training human personnel to use the instrumentation, and the potential need to use a separate room in the animal facility to set up the system. However, these are likely to be outweighed by the benefit of using an approach that offers a more sensitive and objective method to detect disease severity. In addition, the IRAMS can be utilized to perform mechanistic studies to determine the role of molecules/genes/pathways in disease pathogenesis. Thus, IRAMS provides an automated, unbiased, and efficient system to measure disease in a preclinical model of MS, with the potential to be used in other disease models. Additionally, it can be used as a tool to test the therapeutic efficacy of novel MS drugs, which would provide long-term benefits for both scientists studying EAE/MS and patients with the disease.

## Methods

### Mice

Human leukocyte antigen (HLA)-DR3.DQ8 double-transgenic [DQ8 (DQA1*0103, DQB1*0302)-DR3 (DRB1*0301] mice were generated as previously described^14,21^. These mice lack endogenous mouse major histocompatibility complex (MHC) class II genes (AE^−/−^). For simplicity, these mice will be referred as HLA-DR3.DQ8 transgenic mice (Tg) in the text. Eight- to twelve-week-old male and female SJL/J (RR-EAE) and HLA-DR3.DQ8 (Tg) mice were used for the study. Mice were bred and maintained in the University of Iowa animal facility in accordance with NIH and institutional guidelines. All experiments were approved by the Institutional Animal Care and Use Committee at the University of Iowa, Iowa City.

### Disease induction

To induce disease, 8–12 week-old HLA-DR3.DQ8 Tg mice were immunized subcutaneously (s.c) in both flanks with 25 μg of PLP_91–110_ emulsified in CFA containing MTb (400 μg/mice) (Becton, Dickinson and Company, Sparks, MD, USA). Pertussis toxin (PTX; 80 ng) (Sigma Chemicals, St. Louis, MO, USA) was injected intraperitoneally (i.p.) at day 0 and 48 h post immunization, i.e. day 2. Mice were observed daily for clinical symptoms. Disease severity was scored as follows: 0, normal; 1, loss of tail tone; 2, hind limb weakness; 3, hind limb paralysis; 4, hind limb paralysis and forelimb paralysis or weakness; 5, moribund/death. A control group of mice received either PLP_91–110_ plus Incomplete Freund′s adjuvant (IFA) or CFA containing MTb (400 μg/mice), in phosphate buffer saline (PBS) subcutaneously in both flanks. PTX was injected i.p. at day 0 and 48 h post-immunization. To induce relapsing–remitting EAE, 8–12-week-old SJL/J mice were immunized s.c. in both flanks with 100 μg of PLP_139–151_ in CFA containing 200 µg of MTb^22^.

### Disease Activity Monitoring

Locomotory activity of mice was measured using an Opto-M4 multi-channel **A**ctivity **M**onitoring **S**ystem (IRAMS) (Columbus Instruments International, Columbus, OH). Approximate cost of IRAMS is provided as a supplementary data (Supplemental Fig. S1). The IRAMS is able to measure activity in normal mouse or rat cages as opposed to special acrylic boxes using a sensor spaced 4 inches apart with a beam diameter of 0.125 inches and a beam scan rate of 160 Hz. Sensors were screwed on a bracket to allow flexibility for different height adjustments. Cages were placed between these brackets and taken out for routine maintenance such as bedding, food, and water changes (Supplemental Fig. S1). Discrete horizontal (X-axis) and vertical movements (Z-axis) were quantified by the system by tabulating the number of projected infrared beam interruptions at specified time intervals using MDI Software (Columbus Instruments International, Columbus Ohio). Sensors can be adjusted at specified heights to measure horizontal movement (X-axis) or vertical movement/mouse rearing (Z- axis) or both. Thus, two sensors can be added to one bracket to measure both X- and Z-axis activity or just one sensor at specific height to measure either X- or Z-axis activity.

### Treatment of HLA-DR3.DQ8 transgenic mice with Copaxone

We used two protocols for Copaxone (Copaxone, GA, Teva Neuroscience) treatment: prophylactic and therapeutic. In prophylactic setting HLA-DR3.DQ8 Tg mice were immunized with either 2 mg Copaxone or PBS (control group) in 200 μl IFA 10 days before induction of EAE^13^. For the therapeutic protocol, mice received 1^st^ dose of Copaxone 10 days after induction of EAE^13^. Immunized mice were evaluated for EAE disease using the standard EAE scoring method^13^and the IRAMS.

### Statistical analysis

When comparing average clinical EAE scores and analyzing vertical activity, differences between groups were assessed by one-way ANOVA followed by Dunnett’s multiple comparisons test and by the Mann–Whitney rank-sum test when comparing only two groups. Average clinical EAE scores, cumulative EAE scores and vertical activity scores were analyzed by multiple t tests that were calculated using the Holm-Sidak, Benjamini, Krieger, and Yekutieli; Wech’s correction; Kolmogorov-Smirnov method. Statistical analyses were done with GraphPad Prism 7 (GraphPad Software, La Jolla, CA). A value of p ≤ 0.05 was considered significant.

## Supplementary information

Supplementary Figure S1 and S2

## Data Availability

The datasets generated during and/or analysed during the current study are available from the corresponding author on reasonable request.
