# Supplementary material for: Scoring disease in an animal model of multiple sclerosis using a novel infrared-based automated activity-monitoring system
Source: Sci Rep. 2019 Dec 16;9:19194. doi: 10.1038/s41598-019-55713-7 (PMC6915774; doi:10.1038/s41598-019-55713-7)
Supplement: Supplementary file 1 — Supplementary Figure S1 and S2 [file 41598_2019_55713_MOESM1_ESM.pdf]

**Scoring disease in an animal model of multiple sclerosis using a novel infrared-based automated activity-monitoring system**

Shailesh K. Shahi[1], Samantha N. Freedman[1,2], Rachel A. Dahl[4], Nitin J. Karandikar[1,2,3], and Ashutosh K. Mangalam[1,2,3]*

Department of Pathology[1], Interdisciplinary Graduate Program in Immunology[2], and Graduate Program in Molecular Medicine[3], Carver College of Medicine[4], University of Iowa, Iowa City, IA-52242

***Corresponding Author**
Ashutosh K. Mangalam, Ph.D.
Department of Pathology,
University of Iowa Carver College of Medicine,
25 S. Grand Avenue, 1080A ML
Iowa City, IA 52242
Phone: 1-319-335-8558
Fax: 1-319-384-0848
Email. ashutosh-mangalam@uiowa.edu
**ORCID 0000-0002-9926-2531**

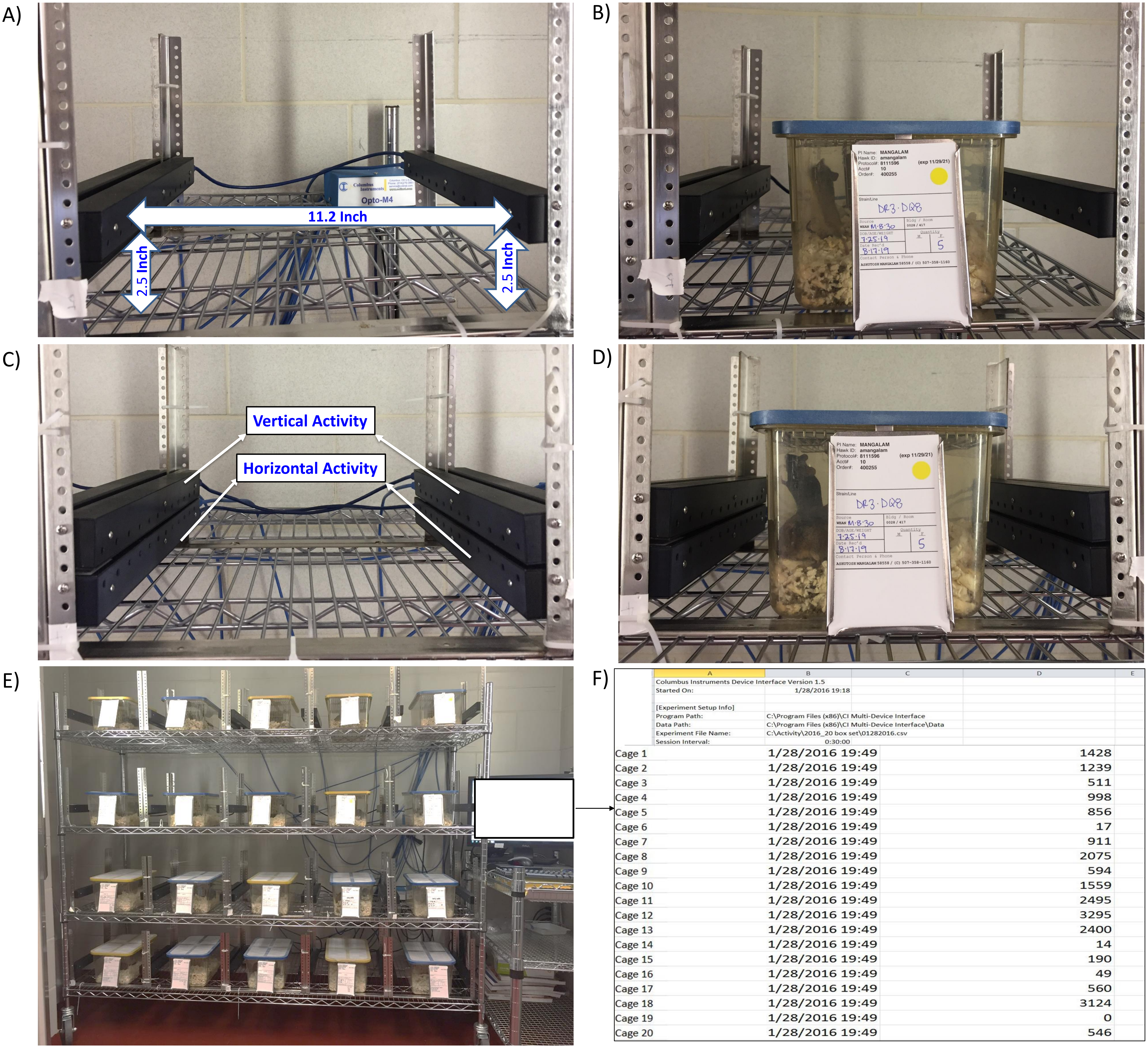


| | A | B | C | D | E |
|---|---|---|---|---|---|
| | Columbus Instruments Device Interface Version 1.5 | | | | |
| | Started On: | 1/28/2016 19:18 | | | |
| | | | | | |
| | [Experiment Setup Info] | | | | |
| | Program Path: | C:\Program Files (x86)\CI Multi-Device Interface | | | |
| | Data Path: | C:\Program Files (x86)\CI Multi-Device Interface\Data | | | |
| | Experiment File Name: | C:\Activity\2016_20 box set\01282016.csv | | | |
| | Session Interval: | 0:30:00 | | | |
| Cage 1 | | 1/28/2016 19:49 | | 1428 | |
| Cage 2 | | 1/28/2016 19:49 | | 1239 | |
| Cage 3 | | 1/28/2016 19:49 | | 511 | |
| Cage 4 | | 1/28/2016 19:49 | | 998 | |
| Cage 5 | | 1/28/2016 19:49 | | 856 | |
| Cage 6 | | 1/28/2016 19:49 | | 17 | |
| Cage 7 | | 1/28/2016 19:49 | | 911 | |
| Cage 8 | | 1/28/2016 19:49 | | 2075 | |
| Cage 9 | | 1/28/2016 19:49 | | 594 | |
| Cage 10 | | 1/28/2016 19:49 | | 1559 | |
| Cage 11 | | 1/28/2016 19:49 | | 2495 | |
| Cage 12 | | 1/28/2016 19:49 | | 3295 | |
| Cage 13 | | 1/28/2016 19:49 | | 2400 | |
| Cage 14 | | 1/28/2016 19:49 | | 14 | |
| Cage 15 | | 1/28/2016 19:49 | | 190 | |
| Cage 16 | | 1/28/2016 19:49 | | 49 | |
| Cage 17 | | 1/28/2016 19:49 | | 560 | |
| Cage 18 | | 1/28/2016 19:49 | | 3124 | |
| Cage 19 | | 1/28/2016 19:49 | | 0 | |
| Cage 20 | | 1/28/2016 19:49 | | 546 | |

**Supplementary Figure S1**: **Schematic presentation of the IRAMS instrumental setup.** IRAMS is a multi-channel activity monitoring system that is placed outside of a standard mouse cage and uses sensors spaced 4 inches apart, with a beam diameter of 0.125 inches and beam scan rate of 160 Hz. Sensors can be arranged at different heights to enable experimental flexibility. The device measures the number of discrete horizontal (X-axis) and vertical movements (Z-axis) by tabulating the number of projected infrared beam interruptions at a specified time interval using MDI Software (Columbus Instrument, Columbus Ohio). A) Set up of single infrared beam emitter (right side) and corresponding detector (left size). B) A mouse cage between single infrared beam emitter and corresponding detector to measure vertical activity. C) Set up of two infrared beam emitters placed at different height (right side) and corresponding detector (left size) to measure both vertical and horizontal activity. D) A mouse cage between two infrared beam emitters placed at different height and corresponding detector to measure both vertical activity and horizontal activity. E) IRAMS with 20 cages of mice to measure vertical activity. F) Numbers of projected infrared beam interruptions at a specified time interval using MDI Software. The total cost of IRAMS to measure vertical activity in 20 cages was $15,000 (based on 2015 price) with no additional service costs. We pay $125/month rent to animal facility to house and use the room. Same system can be use to detect both horizontal and vertical activity in 10 cages.

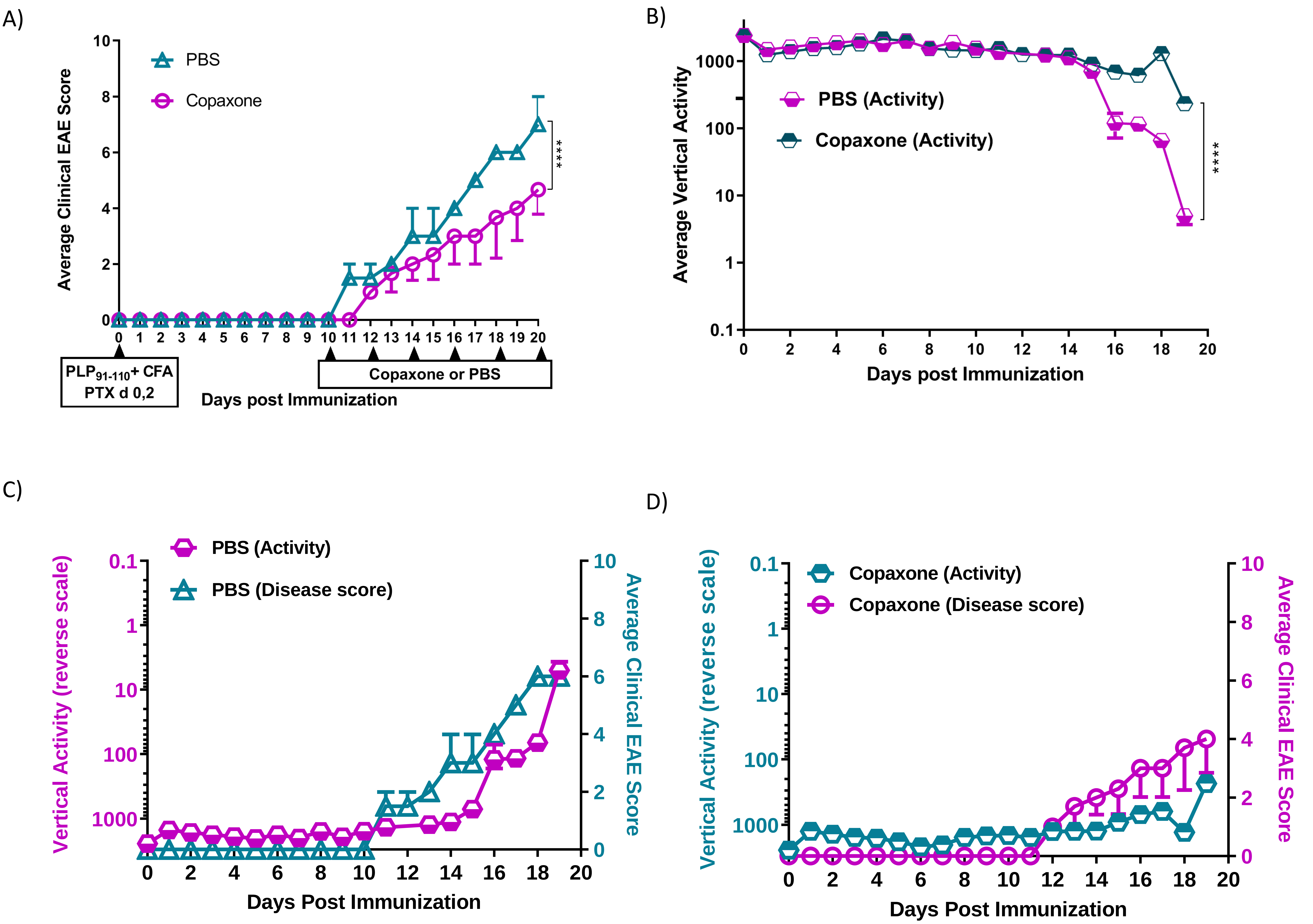


**Supplementary Figure S2: Changes in mouse activity can detect the effects of therapeutic treatment of Copaxone (Glatiramer acetate-GA, Teva Neuroscience) in mice induced with EAE. A)** Average clinical EAE score of HLA-DR3.DQ8 Tg mice immunized on day 0 with $PLP_{91\text{-}110}$ with CFA/PTX and treated with Copaxone or PBS on day 10. Mice were assessed daily using standard 10-point EAE disease scoring for 20 days post-immunization. **B)** Average spontaneous vertical activity of HLA-DR3.DQ8 Tg EAE mice treated with Copaxone or PBS as in A. Measurements of daily average nocturnal activity were obtained up to day 19. **C)** Average clinical EAE score (10-point EAE disease scoring) (right Y-axis) and spontaneous vertical activity (inverted left Y-axis) of mice in A that were only treated with PBS. **D)** Average clinical EAE score (10-point EAE disease scoring) (right Y-Axis) and spontaneous vertical activity (inverted left Y-axis) of mice in A that were only treated with Copaxone. *p*-values for average clinical EAE score (A) and vertical activity analysis (B) determined by one-way ANOVA with Dunnett's multiple comparisons test. **** indicates $p<0.0001$, when compared to the PBS-challenged group.
